# Estimation of Lower Extremity Muscle Activity in Gait Using the Wearable Inertial Measurement Units and Neural Network

**DOI:** 10.3390/s23010556

**Published:** 2023-01-03

**Authors:** Min Khant, Darwin Gouwanda, Alpha A. Gopalai, King Hann Lim, Chee Choong Foong

**Affiliations:** 1 School of Engineering, Monash University Malaysia, Subang Jaya 47500, Malaysia; 2 Department of Electrical & Computer Engineering, Curtin University Malaysia, Miri 98009, Malaysia; 3 Sunway Medical Centre, Petaling Jaya 47500, Malaysia

**Keywords:** inertial sensor, muscle activity, EMG, neural network, long short-term memory

## Abstract

The inertial measurement unit (IMU) has become more prevalent in gait analysis. However, it can only measure the kinematics of the body segment it is attached to. Muscle behaviour is an important part of gait analysis and provides a more comprehensive overview of gait quality. Muscle behaviour can be estimated using musculoskeletal modelling or measured using an electromyogram (EMG). However, both methods can be tasking and resource intensive. A combination of IMU and neural networks (NN) has the potential to overcome this limitation. Therefore, this study proposes using NN and IMU data to estimate nine lower extremity muscle activities. Two NN were developed and investigated, namely feedforward neural network (FNN) and long short-term memory neural network (LSTM). The results show that, although both networks were able to predict muscle activities well, LSTM outperformed the conventional FNN. This study confirms the feasibility of estimating muscle activity using IMU data and NN. It also indicates the possibility of this method enabling the gait analysis to be performed outside the laboratory environment with a limited number of devices.

## 1. Introduction

Inertial measurement unit or IMU has been widely viewed as an economical and practical alternative to the optical motion capture system. A typical optical motion capture involves the placement of numerous reflective markers on anatomical landmarks to measure the movements of lower extremity segments—pelvis, foot, shank and thigh. With the use of IMU, the movement of these segments can be collected by using a limited number of wearable sensors. These can be placed and aligned on the lateral or anterior side of the leg to obtain the kinematics of foot, shank and thigh during walking. The sensors are small and light and can capture human motion outside a laboratory environment. Several studies have demonstrated the accuracy and reliability of the IMU for gait analysis [1,2].

The IMU can be used to derive other valuable information, such as the spatial and temporal gait parameters [3,4]. It can also be used to evaluate and diagnose abnormal gait [5,6] and to identify ageing-related physiological changes [6,7]. Other studies show that IMU alone can be used to perform inverse dynamics analysis to estimate joint moment and ground reaction force in gait [8,9].

A more comprehensive gait analysis involves the use of electromyograms (EMG). Measuring muscle activity using EMG is not a trivial task. Two to three electrodes per muscle must be accurately placed around the muscle to characterize its behaviour. This means that a total of 10 to 15 electrodes have to be placed around the thigh to record the dynamics of the thigh muscle. Surface EMG (SEMG) is a widely adopted measurement technique, but it has several drawbacks. Among them is crosstalk, which happens when SEMG detects the myoelectrical activity of the neighbouring muscle. Moreover, patients often have muscle deformities, which makes it more challenging to obtain accurate readings. Regardless of whether a wireless or wired EMG, a large number of electrodes must be placed on the body. This may hinder the subject from walking naturally, consequently creating gait data that do not represent the actual walking pattern. 

Few studies have attempted to use kinematics and kinetic data to estimate muscle activity in gait. In [10], musculoskeletal modelling and a simulation tool was used to estimate muscle activity. In [11], a feedforward nonlinear autoregressive model with exogenous (NARX) and joint kinetics and kinematics data were proposed to estimate gastrocnemius and tibialis anterior muscle activities. Both studies demonstrated the feasibility of using joint kinematics and kinetics to estimate muscle behaviour. However, they used an optical motion capture system and force place to capture gait data, which can be laborious and time-consuming. Moreover, the gait can only be quantified in a well-controlled environment.

The rapid growth of artificial intelligence, particularly neural network (NN) and its broader adoption in gait analysis can potentially offer means to overcome these limitations. One common use of NN in this field is to identify gait events and gait phases. In [12], IMU was coupled with Long Short-Term Memory (LSTM) to identify the timings of toe-off, heel-strike and mid-swing. In other studies, Deep Convolutional Neural Network (DCNN) was used to detect and classify gait phases in stroke patients [13] and on different terrains [14]. Another widespread use of NN is estimating kinematics or kinetics characteristics of the lower extremity during walking. For instance, Feedforward Neural Network (FNN) together with IMU was used to estimate knee flexion and adduction moment with correlation coefficient (*r*) above 0.69 for walking [15]; Convolutional Neural Network (CNN) and LSTM with lower body joint kinematics were used to estimate muscle forces with *r* greater than 0.83 and joint reaction forces with *r* greater than 0.93 [16]; and CNN, LSTM and CNN-LSTM with EMG were used to estimate joint angles during walking with *RMSE* below 5% [17]. 

Despite the extensive use of NN and IMU to characterize human gait, current literature search suggests that there has not been any attempt made to estimate lower extremity muscle activity using these approaches. Therefore, this study aims to leverage the positive attributes of NN and IMU to estimate muscle activity in human gait. It has two objectives: (1) To create two neural network models i.e., FNN and LSTM to estimate muscle activity using IMU data; (2) To evaluate and compare the performance of the models using standard measures such as normalized Root Mean Square Error (*nRMSE*), correlation of coefficient (*r*) and peak muscle contraction. The outcome of this work can deliver several benefits and overcome the limitations of existing methods. It can reduce the number of modality/device and electrodes attached to the lower extremity and their associated costs, making it more convenient and affordable. It can provide a more extensive overview of gait quality with minimal information. It also enables the gait analysis to be conducted anywhere outside the conventional laboratory environment. Lastly, the use of a small number of compact and light measuring devices will not disrupt the natural gait, thus allowing for a more accurate and reliable gait representation to be recorded and analyzed. 

## 2. Methods

### 2.1. Gait Dataset

This study used an online dataset reported in [18]. It contains walking data collected from 13 healthy male individuals and 9 healthy female individuals (Age: 18–35 years old, height: 1.5–1.8 m, weight: 52–96 kg) walking at three different speeds for ten trials in four different conditions: level ground, ramp, stairs and treadmill. It has two types of data: IMU data and EMG data. The IMU data contain the 3D angular velocity and acceleration of the foot, shank, thigh and trunk. The EMG data contain the muscle activity of gluteus medius, right external oblique, semitendinosus, biceps femoris, rectus femoris, vastus lateralis, vastus medialis, soleus, tibialis anterior and gastrocnemius. The right external oblique muscle is excluded from this study because it is part of the abdominal muscle. This work only focuses on level ground walking regardless of the walking speed; thus, the other data were ignored. The data from 3 subjects were excluded because they contain more than 4 inconsistent muscle activities, possibly due to crosstalk errors during data collection.

### 2.2. Data Processing

The EMG data were pre-processed following the International Society of Electrophysiology and Kinesiology (ISEK) standards [19]. First, Fast Fourier transform (FFT) was used to obtain the EMG power spectrum. It was found that the primary signals lie between 20 Hz and 400 Hz. Therefore, a Butterworth bandpass filter was applied to reduce the noise. Second, the filtered data were rectified. Third, another FFT was performed on the rectified data to obtain the appropriate cut-off frequency to smoothen the signal. A Butterworth low-pass filter with a cut-off frequency of 8 Hz was selected and applied to the rectified data. Next, an EMG envelope was created to obtain the muscle activation profile. 

**Figure 1:**
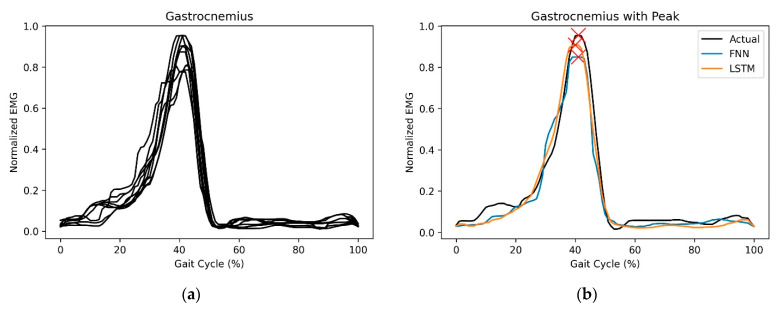
(**a**) The normalized EMG data of glutes medius in several gait cycles; (**b**)The peak muscle contraction of actual and predicted EMG of gastrocnemius in one gait cycle.

In the subsequent step, the EMG data were segmented on a stride-to-stride basis. The timing of the heel strike given in the dataset was used to define the start and end of one stride (one complete gait cycle). The segmented data were then time-normalized to 101 data points, representing the percentage of the gait cycle. The median filter and min-max normalization were then applied sequentially. The median filter is a nonlinear digital filter and is good at removing impulsive noise [20]. The min-max normalization, as defined in (1), creates an array of 101 data points with values ranging between 0 and 1.

(1) $$y_{norm}\left(i\right)=\frac{y\left(i\right)-y_{min}}{y_{max}-y_{min}}$$

where $y_{norm}$ is the normalized data, *y* is the original data, $y_{min}$ is the minimum of the data, $y_{max}$ is the maximum of the data and *i* is the number of data points, *i* = 0, 1, 2, … 100. A sample of processed EMG data is shown in (Figure 1a). The amplitude and timing of the peak muscle contraction (Figure 1b) were identified in every gait cycle to evaluate the performance of the NN in estimating the muscle behaviour.

**Figure 2:**
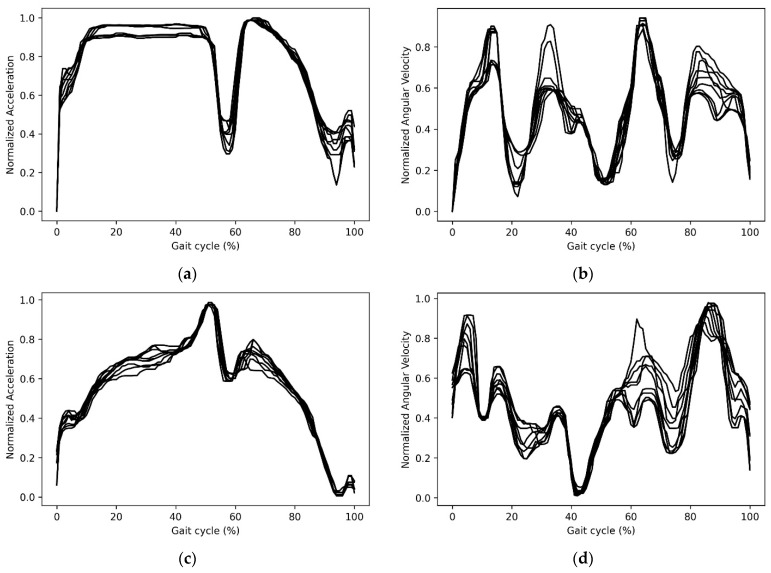
A sample of IMU data. (**a**) Foot x-axis acceleration (**b**) Trunk y-axis angular velocity (**c**) Shank z-axis Acceleration (**d**) Thigh x-axis angular velocity.

The IMU data were processed in the same way as the EMG data, segmented on a stride-to-stride basis and time normalized to 101 data points. The data were then filtered using a median filter and min-max normalized. A sample of the processed IMU data is shown in Figure 2.

### 2.3. Neural Network

**Figure 3:**
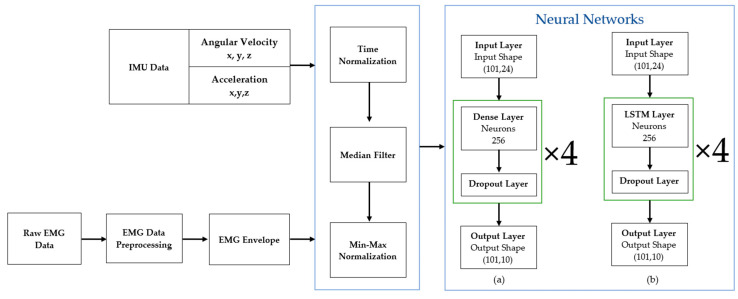
Data pre-processing and the neural network architectures of (**a**) FNN (**b**) LSTM models.

Two NN models were developed here. The first model is an FNN with 1 input layer, 1 output layer, 4 hidden (dense fully connected) layers with 256 neurons each and a drop-out layer between each layer. The input features are arranged in a 2D array that cascades the normalized 3D acceleration and 3D angular velocity of the trunk, thigh, shank and foot in one gait cycle. The target output is 1D normalized EMG data for each individual muscle. The layout of this model is shown in Figure 3a.

The LSTM has similar architecture as the FNN. The only difference between them is that instead of having the dense hidden layer, it has a LSTM hidden layer, as illustrated in Figure 3b. In traditional FNN, the information only flows in one direction—from input to output without any feedback [21]. This means that FNN is only capable of learning linearly separable problems. On the other hand, LSTM can transmit the output backward as the input, therefore LSTM can learn from experience of the process, classify and predict time-series data and remember values for a long time [22]. Both models were developed using TensorFlow. The proposed methodology is summarized in Figure 3.

FNN and LSTM use the same ‘tanh’ as the activation function in the hidden layers. They use the same ‘sigmoid’ as the output layer activation function. Both models used Mean Square Error (MSE) as the loss function and were optimized using Adam optimizer.

The input features and target outputs were split, as shown in Table 1. One random subject data was excluded to be used as an unseen subject test data. The remaining data which has 5440 gait cycles were randomized and divided into 3 groups—training, validation and testing with a ratio of 80:15:5, respectively. 

### 2.4. Validation

A series of measures was used to determine the differences between the predicted and actual muscle activities. Among them are *nRMSE* and *r*, as defined in (2) and (3), respectively.

(2) $$nRMSE=\frac{1}{X_{max}-X_{min}}\sqrt{\frac{1}{n}\sum_{i=1}^{n}{\left(X_{i}-Y_{i}\right)}^{2}}$$

(3) $$r_{XY}=\frac{con\left(X,Y\right)}{\sigma_{X}.\sigma_{Y}}=\frac{\sum_{i=1}^{n}\left(X_{i}-\bar{X}\right).\left(Y_{i}-\bar{Y}\right)}{\sqrt{\sum_{i=1}^{n}{\left(X_{i}-\bar{X}\right)}^{2}}.\;\sqrt{\sum_{i=1}^{n}{\left(Y_{i}-\bar{Y}\right)}^{2}}}$$

where $X_{i}$ are actual EMG at position *i*, $Y_{i}$ are predicted EMG at position *i*, $X_{max}$ is maximum value from actual EMG, $X_{min}$ is the minimum value from actual EMG, $\bar{X}$ is the mean of actual EMG and $\bar{Y}$ is the mean of predicted EMG.

Next, the difference in time and amplitude between the actual and predicted peak muscle contractions were evaluated, as indicated in (4) and (5), respectively.

(4) $$\Delta T_{p}\;\left(\%\right)=T_{x,p}-T_{y,p}$$

(5) $$\Delta E_{p}\;\left(\%\right)=\frac{\left|X_{p}-Y_{p}\right|}{X_{p}}$$

where Δ*T_p_* is the time difference between actual and predicted peak muscle contraction in % of gait cycle, *T_x_*_,*p*_ and *T_y_*_,*p*_ are the times of the actual and predicted peak muscle contractions, respectively, Δ*E_p_* is the percentage difference in amplitude between the actual and predicted peak muscle contraction, *X_p_* is the actual peak contraction and *Y_p_* is the predicted peak contraction. 

Lastly, both predicted and actual muscle activity were plotted together to compare them qualitatively. This involves denormalising and reconstructing the predicted EMG signal back to its original time domain using the heel strike. This is intended to give a more comprehensive outlook of the results, particularly the differences between the predicted and actual muscle behaviours in continuous gait cycles. 

## 3. Results

**Figure 4:**
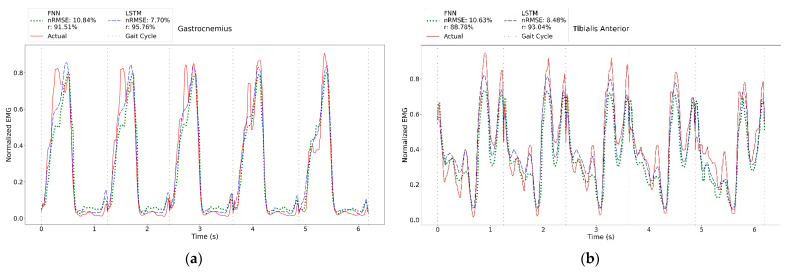

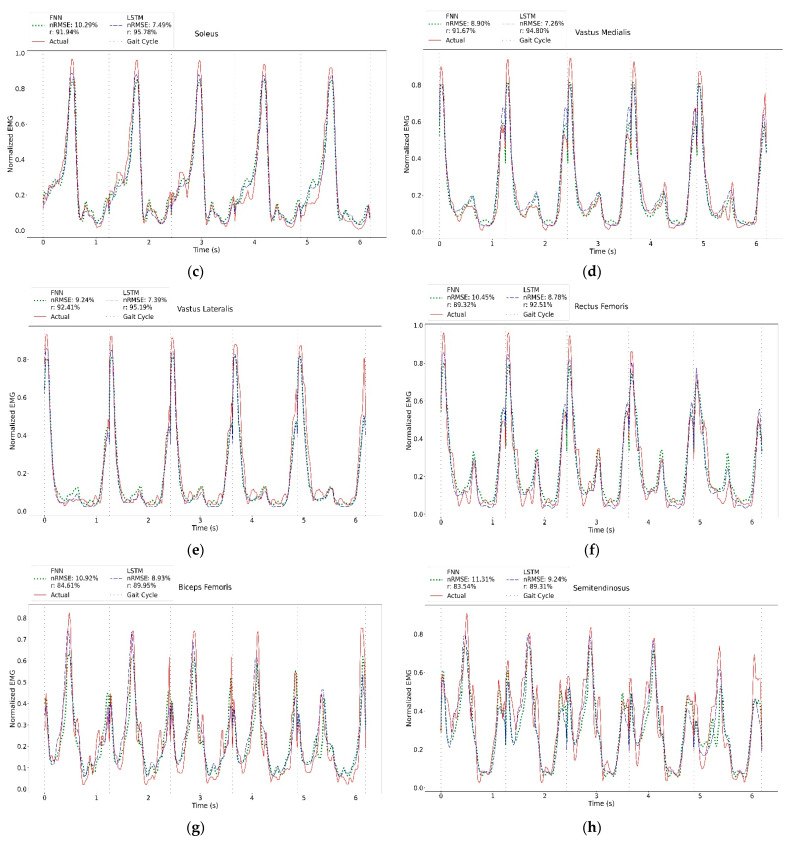

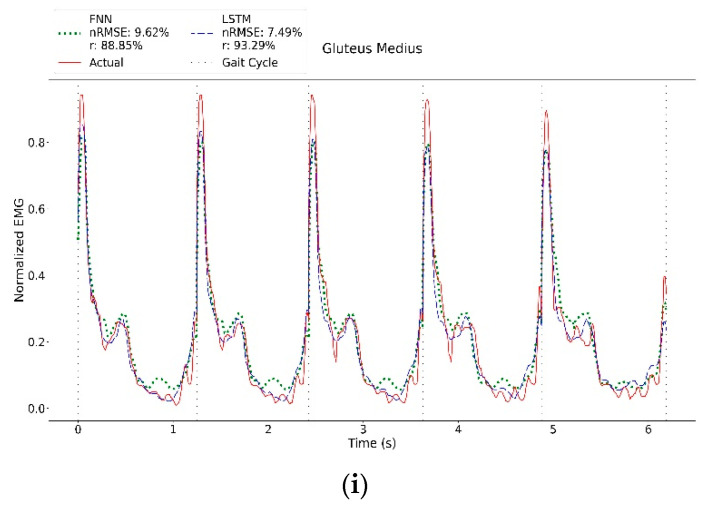
A sample of the actual and predicted muscle activities of the test data of (**a**) gastrocnemius, (**b**) tibialis anterior, (**c**) soleus, (**d**) vastus medialis, (**e**) vastus lateralis, (**f**) rectus femoris, (**g**) biceps femoris, (**h**) semitendinosus, (**i**) gluteus medius.

The average *nRMSE*, *r,* ∆*T_p_* and ∆*E_p_* of the test data are presented in Table 2. Both FNN and LSTM performed well in estimating muscle activities. For example, the average Δ*T_p_* of tibialis anterior muscle is 0.71% and 0.72% of the gait cycle for FNN and LSTM, respectively. The largest Δ*T_p_* was found on the gastrocnemius muscle with an average difference of 2.59% for FNN and 2.40% of the gait cycle for LSTM. FNN and LSTM performed differently when estimating the amplitude of the peak contraction. LSTM can better estimate the peak contraction with an average Δ*E_p_* of less than 20% than FNN with Δ*E_p_* as high as 22%. Despite the discrepancies in peak contraction, both models can estimate muscle activities reasonably well. The estimated EMG waveforms were similar to the actual ones (Figure 4). These results are further corroborated by the small *nRMSE* values and large *r* values. The FNN has *nRMSE* less than 15% and *r* greater than 75%, while LSTM has *nRMSE* less than 10% and *r* greater than 85%. 

**Figure 5:**
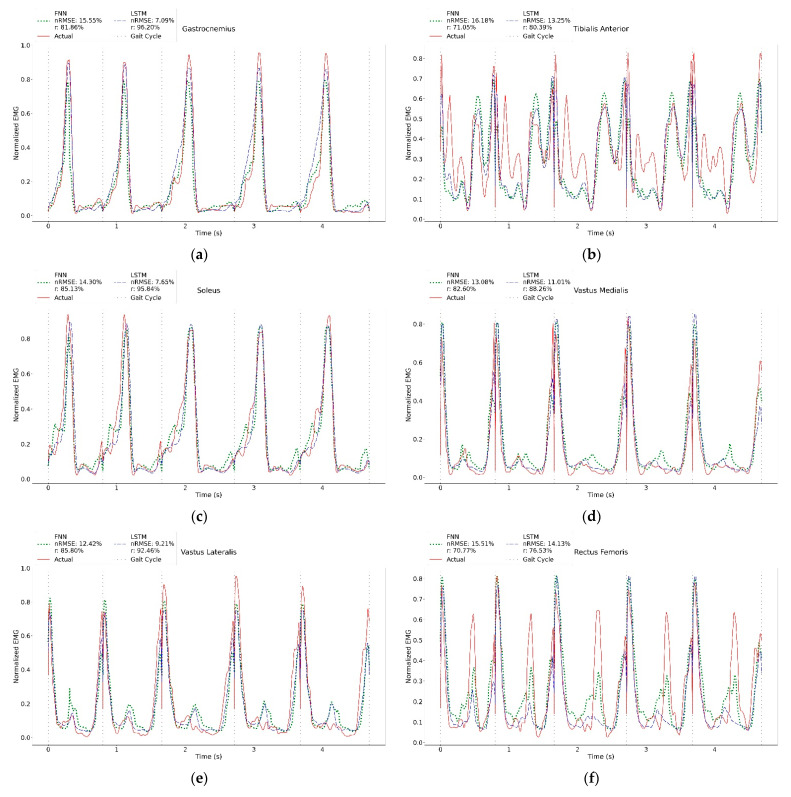

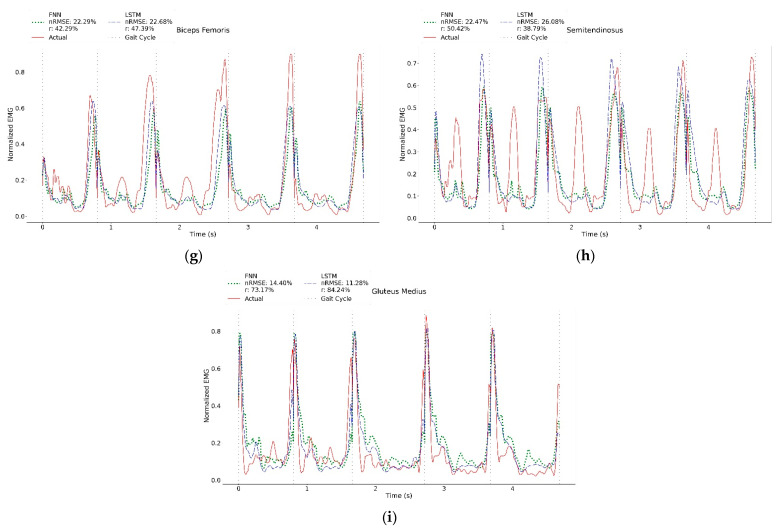
A sample plot of actual vs. predicted muscle activities of the unseen data of (**a**) gastrocnemius, (**b**) tibialis anterior, (**c**) soleus, (**d**) vastus medialis, (**e**) vastus lateralis, (**f**) rectus femoris, (**g**) biceps femoris, (**h**) semitendinosus, (**i**) gluteus medius.

Next, unseen subject data were used to estimate muscle activity to ensure that the model can predict the gait data of a person outside the training and test data. The results are shown in Table 3. Although the average Δ*T_p_* is within an acceptable range, the average Δ*E_p_* are larger than 10%. This is deemed reasonable considering that they are unseen data. Nevertheless, looking into the muscle behaviour in continuous gait cycles (Figure 5), these results are comparable with the literature [23,24]. Both FNN and LSTM can estimate six muscles with *nRMSE* less than 20% and *r* greater than 70%. Breaking down the LSTM results, it can be observed that there are three muscles (gastrocnemius, soleus and vastus lateralis) with *nRMSE* less than 10% and *r* greater than 90%, three muscles (vastus medialis, tibialis anterior and gluteus medius) with *nRMSE* less 15% and *r* greater than 80% and 1 muscle (rectus femoris) with *nRMSE* less than 20% and *r* greater than 75%. The two remaining hamstring muscles (biceps femoris and semitendinosus) performed the worst (*nRMSE* greater than 20% and *r* less 50%). On the other hand, FNN has four muscles with *nRMSE* less than 15% and *r* greater than 80% and two muscles with *nRMSE* less than 20% and *r* greater than 70%. Other similar results can be found in Appendix A. 

## 4. Discussion

This study establishes the possibility of using NN and IMU data to estimate muscle activity. The positive outcome of this work suggests that the number of sensing devices can be reduced, which further implies that the time and effort required for gait analysis can be minimized. Instead of the bulky camera systems and EMG, small and light wearable IMUs can be placed on the trunk and limbs to quantify the kinematics of the gait, thereby making out-of-lab gait analysis a reality. This also indicates that future potential research could lead to home-based, inexpensive gait detection and monitoring of people with gait abnormalities, gait deterioration and injuries. The widespread use of IMU in smartphones and wearable devices, such as fitness trackers, means that potentially more health data, such as gait patterns and muscle activities, can be provided to the users.

The application of NN is promising, particularly the LSTM. It produced a *nRMSE* value less than 15% with *r* greater than 75% for seven muscles. The estimation results also align well with the literature [23,24]. This could be attributed to the main characteristic of the LSTM in retaining information for a long period of time, hence it was able to avoid long-term dependency and generate an output response that closely resembles the actual dynamic behaviour of the muscle. However, it requires greater computational effort than FNN. FNN does not need to remember a lot of information, therefore it uses less computational resources and is generally faster than LSTM.

Trinler et al. used a musculoskeletal model with Static Optimization (SO) and Computer Muscle Control (CMC) to estimate muscle activation of gastrocnemius, tibialis anterior, vastus medialis, vastus lateralis and rectus femoris [10]. Although their results are promising, the current study still performs better with *r* as high as 95% on both test data and unseen subject data. One of the main differences between these two studies is that their study used the conventional optical motion capture system, whereas the current study used IMU data. The other difference is that NN relies on the data to learn and estimate muscle activities. A well-represented data is required for NN to produce an accurate and reliable outcome. On the other hand, SO and CMC rely on the anatomical considerations and assumptions made in the musculoskeletal model. SO calculates the muscle activity by considering the muscle tendons to be rigid and ignoring the passive muscle forces [25]. CMC computes muscle activities from joint coordinates using a combination of proportional-derivative (PD) control and SO [26]. 

The current findings are also in agreement with the study reported by Zabre-Gonzalez et al. [11]. Their study proposed using a NARX neural network and kinematics data derived from the motion capture system to estimate the muscle activity of two muscles. While the NN in the current study focuses on using one generalized model to estimate unseen data, their study focuses on personalized models, therefore two models (one model per muscle) have to be created to estimate the muscle activities.

The actual and predicted muscle activities of gastrocnemius, soleus, vastus lateralis and vastus medialis were similar to those in the literature [23,24], as depicted in Figure 5 and Appendix B. However, for some muscles, minor differences were observed. These were expected, particularly when the unseen data were used. For instance, a typical tibialis anterior muscle (Figure 5b) has two main contractions: one occurs between the pre-swing and mid-swing (between 60–80% gait cycle) and another between terminal swing and opposite toe-off (between 90% of the current gait and 10% of the subsequent gait). In some gaits, the EMG captured small muscle contractions during the stance phase. The NN could not predict these accurately, thus negatively affecting the quantitative results. A similar trend was found in gluteus medius (Figure 5i). 

Several muscles, such as rectus femoris (Figure 5f), biceps femoris (Figure 5g) and semitendinosus (Figure 5h), were reported to have speed-dependent features [27,28,29,30]. For instance, the hamstring muscles (biceps femoris and semitendinosus) activate at the end of the gait cycle (peak around 90% gait cycle) [23,24]. However, in some gaits, an additional contraction was found at the stance phase (around 30% gait cycle). This contraction is more significant in slow walks and the amplitude of this peak can sometimes be greater than the actual contraction. Although this component has been described in previous literature [27,28], it has not been thoroughly explored. Despite its occasional prominence, the NN gave less precedence to this feature and was able to predict the actual contraction accurately. Rectus femoris (Figure 5f) muscle activity occurs during the pre- and initial swing phase (around 50% of the gait) [23,24]. At slow walks, this activity can be minimal (almost zero) and its amplitude increases with speed [29,30]. Due to its high dependence on speed, the NN could not reliably predict this feature. By providing speed or time difference as the inputs in future work, the accuracy of the NN could be improved. On the other hand, the NN could accurately predict the peak muscle contraction at the start of the gait (around 0–20% gait cycle). Although this peak is the most prominent and consistent in all gait data, it is considered to be the crosstalk from vastus lateralis [29,30]. Crosstalk is a known limitation of the SEMG and is widely reported in the literature [31]. Since NN relies on the data to produce correct output responses, this crosstalk will always be present in the predicted results.

The main difficulty faced in this study is inter-subject gait variance. Although the EMG data is normalized to mitigate this issue, secondary or minor peak contractions can have different amplitudes. These peaks are hard to predict and the source of error is difficult to identify. In addition, these secondary peaks can be higher than the actual peak, especially in slow walks, where muscles behave differently. As these peaks are inconsistent and vary from subject to subject, NN cannot accurately estimate muscle behaviour. This can be observed in the results of the unseen test data (Table 2). 

Another limitation of this study is that although the total number of gait cycles is large, these data come from a population with a narrow age group between 18 and 35 years old. This could limit the performance when predicting the muscle activities of the elderly and children. The elderly have different gait characteristics, different gait kinematics and kinetics [32,33] and muscle activity [34] compared to healthy young adults. Likewise, children also have distinct walking behaviors as they have altered body mass distribution and proportion [35], gait features [36] and muscle activities [37]. 

Since this is the first attempt to incorporate IMU and NN to estimate muscle activity, several potential improvements can be explored and investigated in the future. Among them is the use of a larger dataset that includes different types of gaits. Feature extraction in time and frequency domains can be proposed too, such as in [17]. Lastly, different neural network models such as Convolutional Neural Network (CNN) and CNN-LSTM [38] can be developed, trained and compared. 

## 5. Conclusions

This study demonstrates the potential of using IMU data and NN to estimate muscle activity. LSTM performed better than FNN. It was able to estimate three muscles with *r* greater than 90% and *nRMSE* less than 10% and seven muscles with *r* greater than 70% and *nRMSE* less than 20% using IMU data as input. This study also shows that minimal number of modalities/sensors can be used to estimate muscle activity: four IMUs that are attached to the foot, shank, thigh and trunk can estimate nine lower extremity muscle activities during walking. IMU offers several advantages over its conventional counterpart. They are portable and inexpensive, thus allowing the gait analysis to be performed anywhere, outside the laboratory. Studies also show that IMU can produce measurements equivalent to the gold standard. With the wide availability of IMU, gait analysis can be performed remotely for diagnosis and patient monitoring, as well as to provide additional health data. The use of NN here also demonstrates the ability of machine learning to handle gait variation, regardless of its inter-subject variation or inter-stride variation. However, the success of NN heavily relies on the data. Therefore, the first stage of future study will be the collection of gait data that involves a wide range of populations, such as the elderly and patients with gait abnormalities, subsequently exploring different feature extraction methods and neural network models.

## Figures and Tables

**Table 1:** Summary of dataset division.

Group	Number of Trials	Number of Gaits
Training	440	4318
Validation	80	818
Testing	30	304
Unseen subject	30	303

**Table 2:** Comparison between actual and predicted muscle activities on test data.

Muscles	FNN				LSTM			
	*nRMSE* (%)	*r* (%)	∆*T_p_* (%)	∆*E_p_* (%)	*nRMSE* (%)	*r* (%)	∆*T_p_* (%)	∆*E_p_* (%)
Gastrocnemius	10.84	91.51	2.59 ± 2.91	12.96 ± 7.08	7.70	95.76	2.40 ± 2.60	9.21 ± 5.88
Tibialis Anterior	10.63	88.78	0.71 ± 2.14	17.47 ± 10.16	8.48	93.04	0.72 ± 2.16	13.73 ± 10.58
Soleus	10.29	91.94	2.13 ± 1.79	9.08 ± 6.61	7.49	95.78	2.11 ± 1.81	10.54 ± 5.58
Vastus Medialis	8.90	91.67	0.93 ± 0.83	12.30 ± 10.56	7.26	94.80	0.86 ± 0.88	9.60 ± 10.23
Vastus Lateralis	9.24	92.41	0.94 ± 0.80	9.18 ± 7.22	7.39	95.19	0.87 ± 0.84	9.02 ± 6.16
Rectus Femoris	10.45	89.32	1.60 ± 1.65	12.88 ± 9.37	8.78	92.51	1.56 ± 1.74	12.37 ± 15.29
Biceps Femoris	10.92	84.61	1.82 ± 1.59	22.33 ± 15.07	8.93	89.95	1.71 ± 1.43	20.35 ± 14.52
Semitendinosus	11.31	83.54	2.43 ± 2.57	20.95 ± 14.87	9.24	89.31	2.31 ± 2.58	18.16 ± 14.30
Gluteus Medius	9.62	88.85	1.31 ± 2.29	18.11 ± 52.10	7.49	93.29	1.20 ± 2.31	15.48 ± 47.72

**Table 3:** Comparison between the actual and predicted EMG for unseen subject data and with other studies.

Muscle	FNN				LSTM				Other Studies	
	*nRMSE* (%)	*r* (%)	∆*T_p_* (%)	∆*E_p_* (%)	*nRMSE* (%)	*r* (%)	∆*T_p_* (%)	∆*E_p_* (%)	*nRMSE* (%) [11]	*r* (%) [10]
Gastrocnemius	15.55	81.86	1.57 ± 1.80	19.36 ± 9.77	7.09	96.20	1.47 ± 1.08	10.84 ± 12.73	11.0	93
Tibialis Anterior	16.18	71.05	3.89 ± 10.20	31.41 ± 32.21	13.25	80.39	1.75 ± 5.50	18.30 ± 43.19	12.6	66
Soleus	14.30	85.13	1.91 ± 1.65	18.32 ± 9.45	7.65	95.84	1.90 ± 1.42	8.24 ± 2.81	-	96
Vastus Medialis	13.08	82.60	0.78 ± 0.62	12.84 ± 22.10	11.01	88.26	1.44 ± 0.65	14.34 ± 24.80	-	60
Vastus Lateralis	12.42	85.80	0.91 ± 0.82	12.13 ± 24.22	9.21	92.46	1.27 ± 0.80	17.53 ± 23.29	-	61
Rectus Femoris	15.51	70.77	1.49 ± 3.21	20.20 ± 35.08	14.13	76.53	1.70 ± 3.52	21.79 ± 39.68	-	54
Biceps Femoris	22.29	42.29	4.17 ± 2.91	24.00 ± 19.82	22.68	47.39	2.43 ± 1.99	36.44 ± 33.66	-	-
Semitendinosus	22.47	50.42	2.62 ± 2.09	23.88 ± 17.96	26.08	38.79	4.52 ± 2.39	24.73 ± 26.10	-	54
Gluteus Medius	14.40	73.17	0.82 ± 2.59	11.64 ± 12.84	11.28	84.24	1.70 ± 2.14	10.59 ± 13.91	-	-

**Figure A1:**
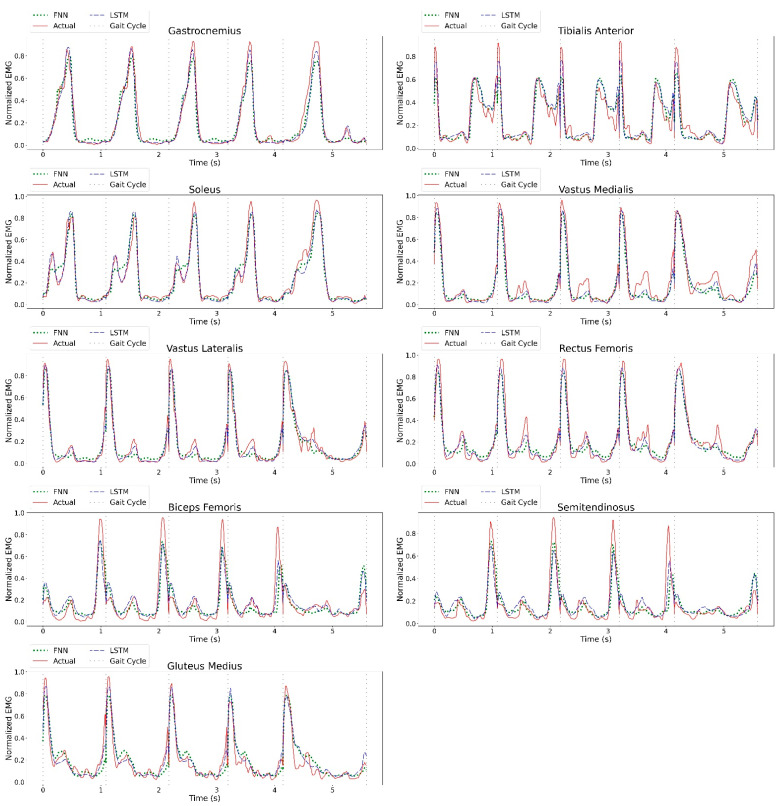
A plot of actual vs. predicted muscle activities of the test data (Sample A).

**Figure A2:**
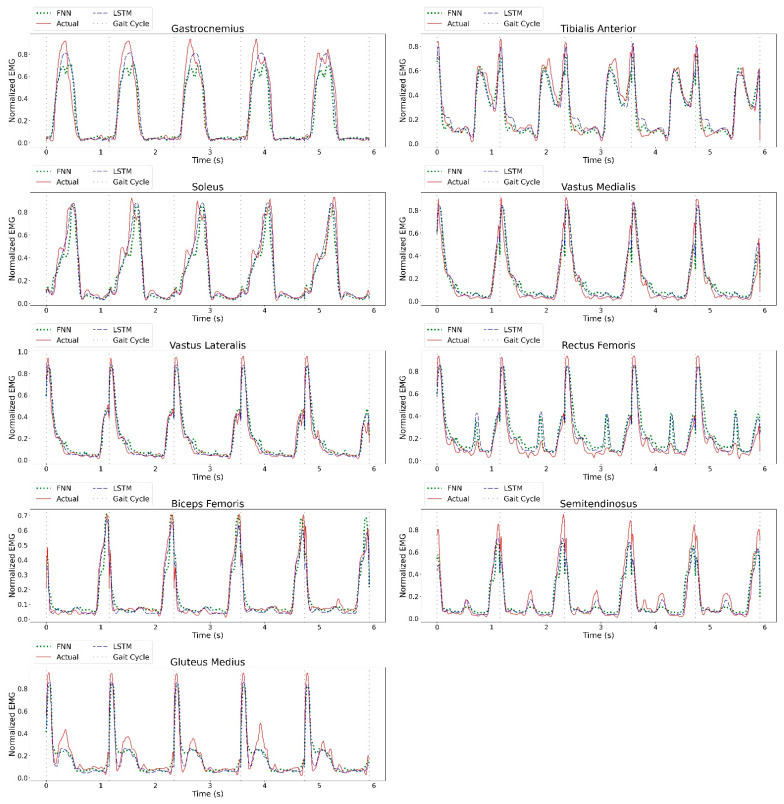
A plot of actual vs. predicted muscle activities of the test data (Sample B).

**Figure A3:**
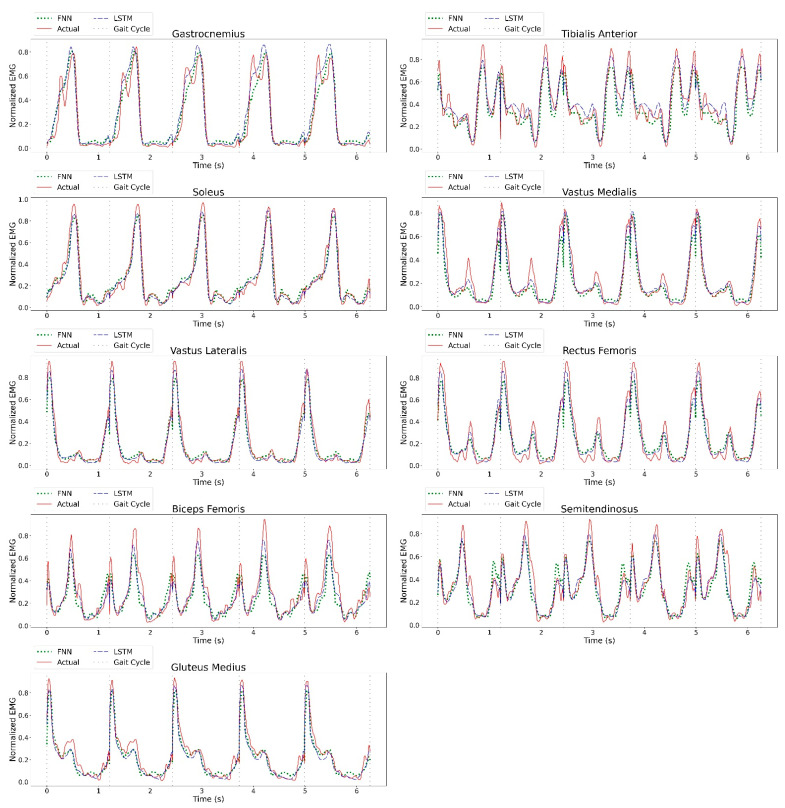
A plot of actual vs. predicted muscle activities of the test data (Sample C).

**Figure A4:**
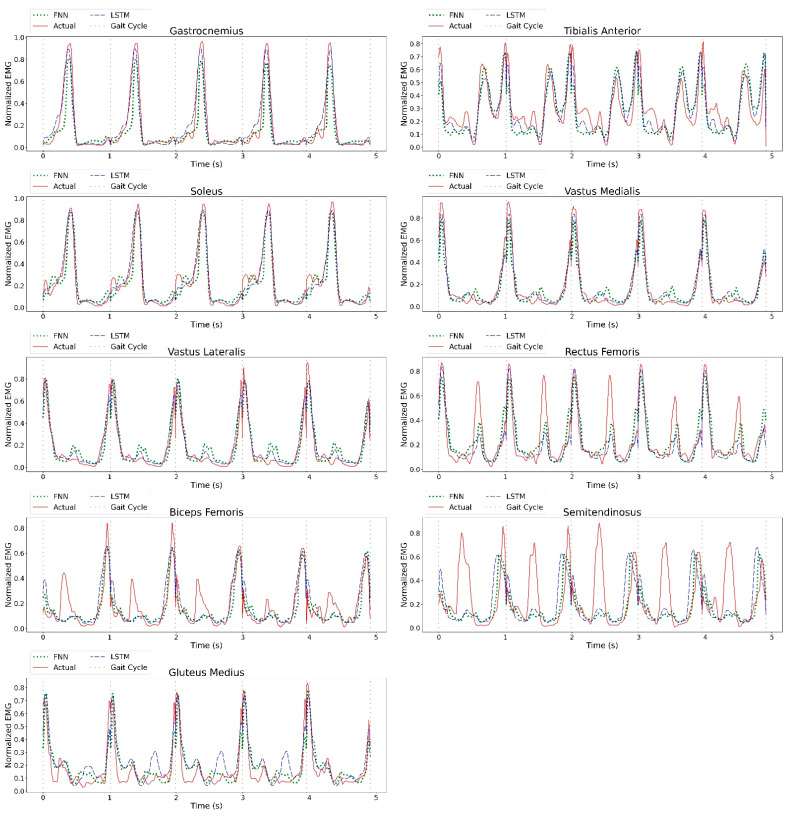
A plot of actual vs. predicted muscle activities of the unseen data (Sample A).

**Figure A5:**
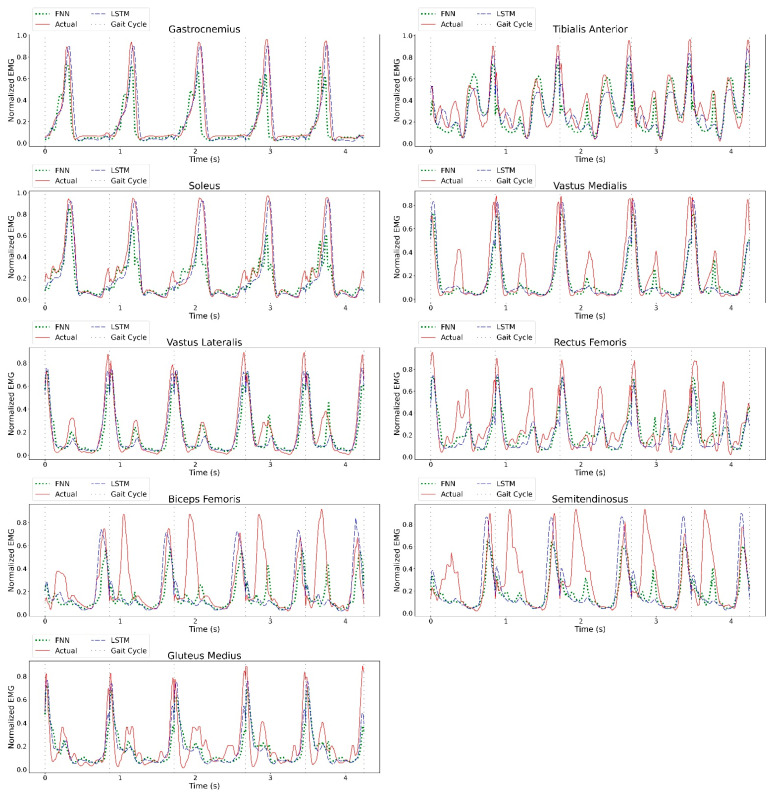
A plot of actual vs. predicted muscle activities of the unseen data (Sample B).

**Figure A6:**
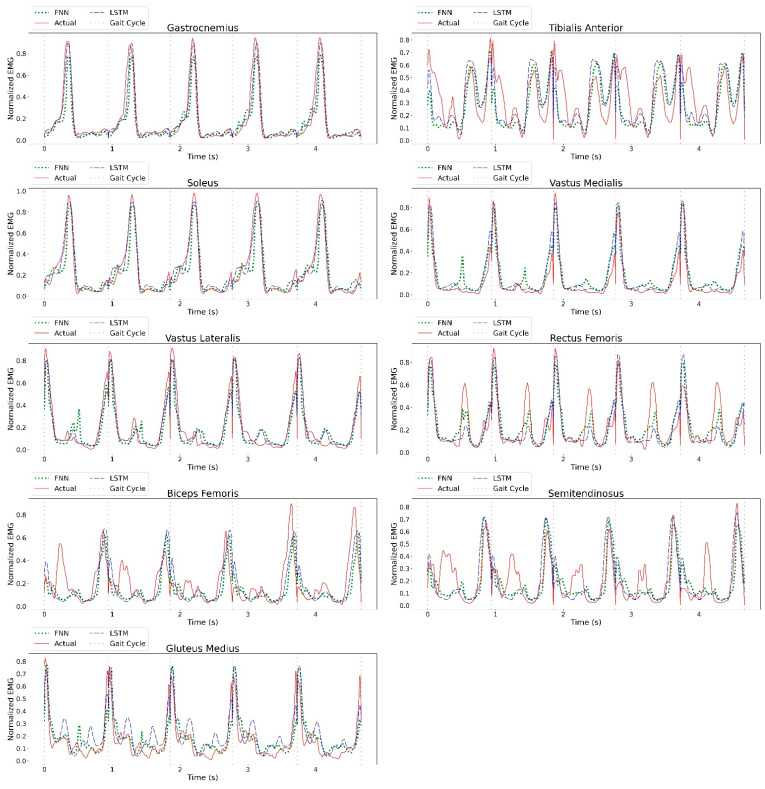
A plot of actual vs. predicted muscle activities of the unseen data (Sample C).

## Data Availability

The original data can be obtained from the open access online dataset by Camargo, et al. [18]. The results of this study can be found in the link below and other data can be provided with reasonable request: https://github.com/mubarakmin/Estimating-Lower-Extremity-Muscle-Activity-in-Gait-using-IMU-and-NN (accessed on 15 November 2022).
